# Severe Periodontal Disease Associated with Long-Term Treatment with Intravenous Immunoglobulin

**DOI:** 10.1155/2014/860804

**Published:** 2014-10-15

**Authors:** Jôice Dias Corrêa, Amanda Leal Rocha, Lidiane Cristina Machado Costa, Denise Travassos, Wagner Henriques Castro, Gustavo Pompermaier Garlet, Rodrigo Santiago Gomez, Antônio Lúcio Teixeira, Tarcília Aparecida Silva

**Affiliations:** ^1^Department of Oral Surgery and Pathology, School of Dentistry, Federal University of Minas Gerais, 31270-901 Belo Horizonte, MG, Brazil; ^2^Special Service for Diagnosis and Treatment in Dentistry (SEDTO), Hospital and Clinics, Federal University of Minas Gerais, 30130-100 Belo Horizonte, MG, Brazil; ^3^Department of Community and Preventive Dentistry, School of Dentistry, Federal University of Minas Gerais, 31270-901 Belo Horizonte, MG, Brazil; ^4^Department of Biological Sciences, Bauru School of Dentistry, University of São Paulo, 17012-901 Bauru, SP, Brazil; ^5^Neurology Service, Hospital and Clinics, Federal University of Minas Gerais, 30130-100 Belo Horizonte, MG, Brazil

## Abstract

Intravenous immunoglobulin (IVIG) is used in the treatment of neuropathy. This case report presents, for the first time, a patient with severe periodontal destruction after chronic therapy with IVIG. The patient reported having extracted his maxillary anterior teeth himself due to high mobility. Clinical examination and radiographic images show a generalized and severe periodontitis. No significant alterations in genetic or microbiological features were observed. The present case suggests that periodontal disease aggravation could be considered a new adverse effect of IVIG therapy. Postulated mechanisms are immune complexes formation, complement activation, and a direct effect in osteoclasts. In conclusion, it is important that patients that will receive IVIG treatment underwent dental evaluation.

## 1. Introduction

Multifocal motor neuropathy (MMN) with conduction block is an acquired immune-mediated demyelinating neuropathy with slowly progressive weakness, without significant sensory involvement [1]. It can be mistaken for amyotrophic lateral sclerosis (ALS), especially if muscle fasciculations are present. Unlike ALS, MMN exhibits asymmetric weakness and affects only the lower motor neuron. The symptoms usually begin in upper limbs [1, 2]. Multiple immunomodulatory and immunosuppressive treatments have been used, but the most promising therapeutic strategy is based on the long-term use of intravenous infusion of immunoglobulins (IVIG). IVIG is the treatment of choice in NMM (evidence level I) [2]. NMM does not respond to corticosteroids and immunosuppressive therapy. Some cases of NMM may respond to cyclophosphamide. However cyclophosphamide toxicity precludes long-term use, which is usually necessary in patients with MMN. Although plasma exchange and prednisone are well-established therapies in other immune-mediated neuropathies, they are not effective in patients with MMN and may even aggravate symptoms [2].

IVIG is a preparation of highly purified immunoglobulin, predominantly composed by IgG subclasses [1]. IVIG treatment has been reported to cause adverse effects, including headache, myalgia, fatigue, diarrhea, blood pressure changes, tachycardia, and anaphylactic reactions [3, 4].

Periodontitis (PD) is a chronic infectious disease characterized by plaque-induced destruction of soft and hard periodontal tissues [5]. In addition to periodontopathogenic microorganisms, genetic (especially linked to host immune response) and environmental factors contribute to cause PD [6]. Observational studies have reported bidirectional associations between PD and various systemic diseases, but the causal relations have not been well established [7]. Some mechanisms proposed to explain these observations are the use of immunomodulatory and immunosuppressive drugs to control systemic diseases that could directly affect periodontium. These medications reduce inflammatory response to bacterial plaque [8], which could impact on oral microbiota load and composition, predisposing the colonization and/or infection with periodontopathogenic microorganisms [8, 9].

This report presents, for the first time, a possible new adverse effect of IVIG treatment, with a patient in chronic use of this medication showing severe and generalized periodontal destruction.

## 2. Case Report

### 2.1. Medical History

A 36-year-old man presented with tooth mobility at Serviço Especial de Diagnóstico e Tratamento em Odontologia (SEDTO) at Hospital das Clínicas, Universidade Federal de Minas Gerais (HC-UFMG). Patient was referred from the Serviço de Neurologia. In 2013, he reported extracting himself his maxillary anterior teeth due to high mobility of those teeth. According to the patient, this dental alteration began after the start of medical treatment with IVIG.

From 2007 patient developed progressive muscle weakness, with difficulties to walk and climb stairs. Clinical examination showed weakness on all four members with hiporreflexia but no changes on sensory function and cranial nerves. An extensive laboratory work-up was performed, including rheumatologic screening (e.g., antinuclear antibodies (ANA), rheumatoid factor, erythrocyte sedimentation speed, and reactive protein C), without any significant finding. Electroneuromyography showed an asymmetric multifocal blocking of axonal and myelinic motor conduction. The patient was then diagnosed with “multifocal motor neuropathy with conduction blocks” and started, in 2007, monthly to use intravenous immunoglobulin (2 mg/kg during five days a month). The evolution of the clinical status (improvement on muscle strength) was considered excellent. During 2009 there was a modification in the therapeutic scheme, with the beginning of monthly cyclophosphamide pulse therapy (1 g/cycle; 10 cycles); however, the patient responded with worse clinical features, which suggested “dependence” of immunoglobulin to control the symptoms, and then the IVIG treatment was restarted.

Recently, the patient has a stable motor status with monthly immunoglobulin scheme.

### 2.2. Dental Analysis

**Figure 1:**
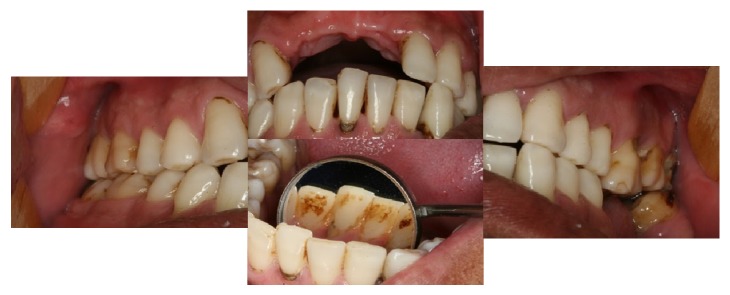
Clinical features of the severe periodontal disease at first consultation. Anterior teeth lost and accumulation of gross calculus and tooth pigmentation.

**Figure 2:**
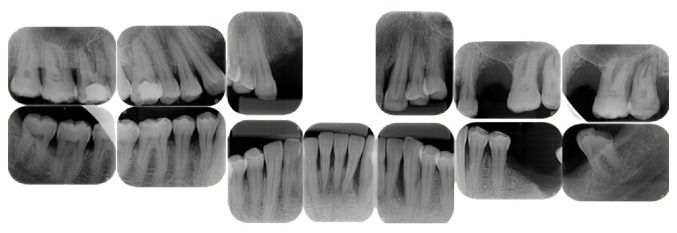
Radiographic features of severe periodontal disease. Generalized vertical and horizontal bone loss.

In April 2013 the patient was sent to dental evaluation after complain of an accentuated dental mobility after the start of therapy with immunoglobulin. Before that, patient did not notice any bleeding or modifications in gingiva. During the anamnesis, the patient reported previous smoking habit, but he stopped since 2007. Upon clinical examination the patient showed dental mobility in several elements, accumulation of gross calculus, and tooth pigmentation (Figure 1). Radiographic examination of the present teeth showed generalized and marked bone loss (Figure 2). The patient was forwarded to the School of Dentistry of UFMG for evaluation and treatment by a periodontist (LMC).

Patient underwent complete periodontal examination, in which bleeding on probing (BOP) in 52% of sites was observed, probing depth (PD) > 4 mm in 37% of all dental sites (Table 1). The maxillary left first molar and the mandibular left second molar presented periodontal pocket with 10 mm of PD and abnormal mobility; thus, the extraction was indicated. Clinical diagnosis was generalized and severe chronic periodontitis.

### 2.3. Microbiological and Genetic Analysis

To better explore mechanisms that could explain the severe periodontal status, subgingival dental plaque samples were collected by sterile endodontic paper points ISO number 40 (Tanariman LTDA, Manacaparu, AM, Brazil). The paper points were inserted in the 5 sites with deepest periodontal pockets and kept there for one minute. Subgingival samples were used for qPCR assays for microorganism detection. Bacterial or viruses DNA was extracted as previously described [10] and analyzed by qPCR using SybrGreen system (Invitrogen, Carlsbad, CA, USA) and specific primers (the primer sequences are depicted in Table 2). The positivity for bacteria or viruses in each sample was determined based on the comparison with positive and negative controls.

*Actinomyces actinomycetemcomitans, Tannerella forsythia*,* and Porphyromonas gingivalis* were found in the subgingival samples (Table 2) and the levels of* A. actinomycetemcomitans, T. forsythia*, and* P. gingivalis* were comparable with that found in chronic periodontitis patients as previously reported [11]. On the other hand,* F. nucleatum*, and* T. denticola* were not detected. To define if medication had impact on whole microbiota we also analyzed the presence of viruses, since studies have shown that herpesviruses may be related to the etiology of aggressive and chronic periodontitis by triggering periodontal destruction or by increasing the risk for bacterial infection [12, 13]. However, there was no DNA detection for viruses Epstein-Barr virus type 1 (EBV-1), Herpes Simplex virus type 1 (HSV-1), or* Human cytomegalovirus* (HCMV) (Table 2).

In order to verify whether the bad periodontal condition was linked to a susceptible background, the patient was typed for HLA molecules [11, 12] and single nucleotide polymorphisms (SNPs) in target genes associated with periodontal disease susceptibility. We analyzed IL1B-3954, IL6-174, MMP1-1607, TNFA-308, IL10-592, and TGFB1-509 SNPs. Only TNFA-308, IL10-592, and TGFB1-509 were polymorphic (Table 2). The genetic analysis also demonstrated that the patient is not carrier of the alleles HLA-DR4 (0404) and HLA-B27 described to be associated with bone resorptive diseases. Altogether, no genetic or microbiological tested parameters seem to explain or be associated with this severe periodontal condition. Therefore, the data reinforce that the present case probably represents a preexistent periodontal disease (periodontitis or gingivitis) aggravated by the use of immunoglobulin.

### 2.4. Periodontal Therapy

The patient was submitted to periodontal therapy which included sessions of supra and subgingival scaling and root planning to control local factors that could worsen periodontal disease. Moreover, extraction of left maxillary first and third molar and left mandibular second molar was done. The patient was forwarded to prosthetic rehabilitation. At present, the patient is stable, in follow-up in the SEDTO at HC-UFMG for periodic periodontal monitoring.

**Figure 3:**
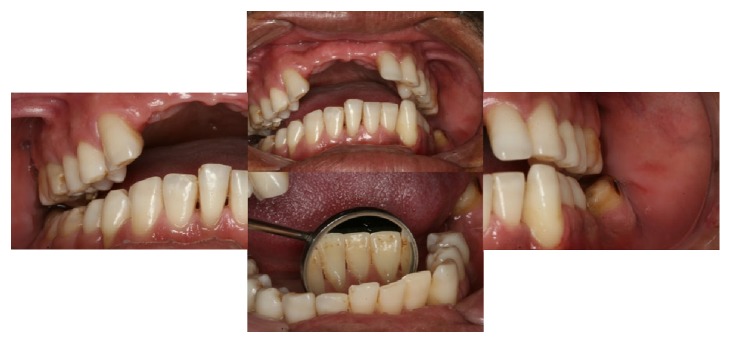
Clinical features after periodontal therapy. Good hygiene without calculus or plaque.

The last periodontal examination, in January 2014, showed that PD was reduced in most of the sites and the BOP was strongly reduced (Table 1). Patient shows satisfactory oral hygiene without plaque and calculus accumulation (Figure 3).

## 3. Discussion

Currently, bidirectional associations of PD with systemic diseases have been reported [7]. In the present case we showed a patient suffering with MMN that reports loss of his anterior teeth after long-term treatment with IVIG. Clinical and radiographic examinations demonstrated a severe and generalized periodontal disease, with clinical attachment loss and bone loss in almost all teeth and spread gingival inflammation. Regardless of the presence of 3 SNPs in genes that regulate immune response (*TNFA*,* IL10*, and* TGFb*), no other genetic or microbiologic analyses were contributing factors to explain this severe disease, reinforcing the association of PD and IVIG treatment.

Despite we have no dental record before IVIG treatment, both medical evaluation and patients' perception consistently reported that oral condition became worse after the beginning of therapy. Therefore, the hypothesis is that the use of immunoglobulin exacerbates the preexistent periodontal condition, remaining undefined if patient had or not alveolar bone loss before using IVIG. This report describes, for the first time, a possible adverse reaction after IVIG treatment. It is necessary to better understand the mechanisms of this association.

Intravenous immunoglobulin is a preparation of highly purified immunoglobulin collected from a large pool of healthy human plasma. Human IVIG contains biologic active IgG and trace amounts of IgA, IgM, CD4, CD8, and human leukocyte antigen molecules [14]. Because IVIG preparations are heterogeneous, it is difficult to determine its exact mechanism of action. It is widely postulated that the efficacy of IVIG therapy is linked to its ability to block Fc receptors, eliminate autoantibodies, modulate cytokine synthesis, inhibit complement, and mediate Fas-Fas ligand interactions [10].

In view of the IVIG mechanism of action it was expected that IVIG treatment might decrease the inflammation in periodontal sites, consequently, diminishing bone and attachment loss. However, in the case reported, we observe a worse of periodontal condition. A possible explanation is the formation of immune complexes, with IgG aggregates, that could lead to activation of complement cascade [14]. Under physiological conditions, the complement is beneficial for host response to destroy and eliminate microbes and dead tissues [15]. However, chronic infections, such as periodontitis, have the tendency to keep the formation of immune complexes and when the immune complexes are not eliminated, the complement cascade became chronically activated, promoting inflammation [16].

Another mechanism related to adverse reactions in IVIG therapy is the formation of oligomeric or polymeric IgG complexes that interact with Fc receptors and trigger the release of inflammatory mediators [3]. The direct ligation of IgG to Fc receptors on immature osteoclast could result in enhanced osteoclast generation and, ultimately, bone destruction. This mechanism was showed in another study using an experimental model of arthritis [17]. Accordingly, there is a report of patients developing arthritis after IVIG therapy [3].

In general, adverse reactions to IVIG therapy are usually minor and occur in no more than 10% of the patients [3, 4]. The most common side effect of IVIG use is acute hypersensitivity, and others include headache, flushing, malaise, chest tightness, fever, chills, myalgia, fatigue, dyspnea, back pain, nausea, vomiting, diarrhea, blood pressure changes, and tachycardia [3, 4]. Even rare, adverse effects could complicate the treatment and worse health quality of the patients. The present case suggests that periodontal disease aggravation could be considered a new adverse effect of IVIG therapy. Postulated mechanisms are immune complexes formation, complement activation, and a direct effect in osteoclasts. In conclusion, it is important that patients that will receive IVIG treatment underwent dental evaluation.

## Figures and Tables

**Table 1:** Clinical features before and after the periodontal therapy.

	Periodontal therapy	
	Before	After
PD (mm)	4.10	2.31
CAL (mm)	4.44	2.60
BOP (%)	51.78	24.0

PD: probing depth; CAL: clinical attachment loss; BOP: bleeding on probing.

**Table 2:** Primer sequences from the genetic and microbiological analysis.

Target	Sense/antisense sequences
*A. actinomycetemcomitans *	ATGCCAACTTGACGTTAAAT
	AAACCCATCTCTGAGTTCTTCT

*P. gingivalis *	TACCCATCGTCGCCTTGGT
	CGGACTAAAACCGCATACACTT

*F. nucleatum *	GCGGAACTACAAGTGTAGAGGT
	GTTCGACCCCCAACACCTAGTA

*T. denticola *	AGAGCAAGCTCTCCCTTACCGT
	TAAGGGCGGCTTGAAATAATGA

*T. forsythia *	AGAGCAAGCTCTCCCTTACCGT
	TAAGGGCGGCTTGAAATAATGA

EBV-1	CCTGGTCATCCTTTGCCA
	TGCTTCGTTATAGCCGTAGT

HSV-1	CGGCCGTGTGACACTATCG
	CTCGTAAAATGGCCCCTCC

HCMV	TGAGCCCGGCGGTGGT
	AGCTCACCGATCACAGACAC

IL1B-3954 SNP	CTCAGGTGTCCTCGAAGAAATC
	GCTTTTTTGCTGTGAGTCCCG

TNFA-308 SNP	AGGCAATAGGTTTTGAGGGCCA
	TCCTCCCTGCTCCGATTCCG

IL6-174 SNP	TTGTCAAGACATGCCAAGTGCT
	GCCTCAGAGACATCTCCAGTCC

IL10-592 SNP	GGTCTCTGGGCCTTAGTTTCC
	AACTTTAGACTCCAGCCACAGA

TGFB1-509 SNP	TTTTGCCATGTGCCCAGTAG
	CACCAGAGAAAGAGGACCAG

MMP1-1607 SNP	TCGTGAGAATGTCTTCCCATT
	TCTTGGATTGATTTGAGATAAGT
